# Iron sourcing strategies and adaptive protein responses to iron deprivation in the intraerythrocytic parasite *Babesia divergens*

**DOI:** 10.1371/journal.pntd.0014861

**Published:** 2026-11-04

**Authors:** Viktoriya Levytska, Jan Mach, Luise Robbertse, Celia W. Goulding, Ronald Malych, Marie Jalovecka, Robert Sutak, Daniel Sojka

**Affiliations:** 1 Department of Disease Vectors and Pathogens, Institute of Parasitology, Biology Centre of the Czech Academy of Sciences, České Budějovice, Czech Republic; 2 Department of Parasitology, Faculty of Science, BIOCEV, Charles University, Vestec, Czech Republic; 3 Departments of Molecular Biology and Biochemistry and Pharmaceutical Sciences, University of California, Irvine, California, United States of America; 4 Faculty of Science, University of South Bohemia, České Budějovice, Czech Republic; Universidade do Estado do Rio de Janeiro, BRAZIL

## Abstract

Iron is indispensable for the survival and pathogenicity of *Babesia* parasites, yet the mechanisms underlying its acquisition remain largely unresolved. In contrast to other apicomplexan parasites, tick-borne *Babesia* species rapidly lose the parasitophorous vacuole following erythrocyte invasion and do not form hemozoin, suggesting the evolution of distinct strategies for iron metabolism. Here, we demonstrate that *Babesia divergens* utilizes a sequential, multi-route iron acquisition system to meet its metabolic demands within host erythrocytes. Kinetic uptake analyses reveal a pronounced preference for ferrous iron (Fe^2+^), which is imported through an active, energy-dependent process supported by extracellular ferric reductase activity that converts host-derived ferric iron (Fe^3+^) into a bioavailable form prior to uptake. Using immunogold transmission electron microscopy, we provide evidence for the active internalization of host transferrin and show that parasite proliferation is dose-dependently inhibited by apo-transferrin, establishing iron as a limiting factor for parasite growth. Quantitative proteomic profiling revealed a limited, stage-specific response to deferoxamine-induced iron deprivation. Three proteins showed statistically significant changes in abundance in free merozoites: the conserved multipass membrane protein Bdiv_019230 increased, whereas the hypothetical protein Bdiv_002300 and the putative serine hydroxymethyltransferase Bdiv_027330 decreased. No proteins reached the statistical significance threshold in intraerythrocytic parasites. These results identify a small set of statistically supported candidates for investigating the response of extracellular *B. divergens* merozoites to iron-restricted conditions.

## Author summary

To survive and proliferate within vertebrate hosts, the intraerythrocytic parasite *Babesia divergens* must obtain iron from the tightly regulated environment of red blood cells. Although iron is essential for parasite growth and survival, iron acquisition and homeostasis in *Babesia* remain largely unexplored. In this study, we investigated iron metabolism in *B. divergens* through a series of complementary approaches.

First, we examined how the parasite takes up iron and demonstrated that reductive iron uptake enables the incorporation of iron into functional protein complexes. Second, we assessed the parasite’s ability to exploit host iron sources and found that transferrin-bound iron supports parasite propagation within erythrocytes. Third, we used proteomic analyses to define how different blood-stage forms of *B. divergens* respond to iron chelation, revealing stage-specific metabolic adaptations to iron limitation. Finally, we identified a highly abundant iron-responsive protein that may contribute to parasite adaptation to iron limitation and other stress conditions.

Together, our results show that *B. divergens* employs flexible and coordinated strategies to acquire and utilize iron within host red blood cells. This work advances understanding of iron-dependent host-parasite interactions and highlights parasite iron metabolism as a promising target for future therapeutic interventions against babesiosis.

## Introduction

*Babesia* spp. are intraerythrocytic apicomplexan protozoan parasites of medical and veterinary significance, responsible for the disease babesiosis across a wide range of mammalian hosts. In animals – particularly in livestock and domestic canines – *Babesia* infections often manifest as acute or chronic hemolytic anemia and, in severe cases, may lead to fatal outcomes. In humans, babesiosis presents as a malaria-like illness, characterized by fever, hemolysis and systemic complications. While often self-limiting in immunocompetent individuals, the infection can be life-threatening in immunosuppressed patients, asplenic individuals or those with underlying comorbidities [1,2].

Iron metabolism within the host is a critical determinant in the establishment and progression of parasitic infections [3]. As an essential micronutrient, iron serves indispensable roles in numerous biological processes that sustain Apicomplexa survival and pathogenicity, particularly in energy production, DNA replication and repair and redox regulation [4–6].

Apicomplexan parasites, such as *Plasmodium*, *Toxoplasma*, *Neospora*, *Babesia*, and *Theileria* rely on specialized organelles for iron processing and iron–sulfur cluster (ISC) biosynthesis [7,8]. The ferredoxin-NADP⁺ reductase (FNR)/ferredoxin (Fd) system in the apicoplast provides reducing power for key pathways such as isoprenoid precursor synthesis and ISC assembly [9,10]. Despite reductive evolution, the mitochondrion retains a functional ISC biosynthesis pathway essential for electron transport and energy metabolism [8,11]. These iron-processing pathways, in combination with iron transport and storage systems, represent metabolic vulnerabilities that could be exploited for antiparasitic drug development [12].

Iron availability profoundly influences cellular physiology and proliferation in diverse microbial and parasitic organisms, with elevated iron levels generally facilitating enhanced metabolic activity, replication and survival [13,14]. Similar mechanisms may also operate in *Babesia*, although they remain less well characterized. Studies have demonstrated that supplying iron in vivo can promote the reproduction of *Babesia microti* [15]. Conversely, apo lactoferrin, has been shown to inhibit the growth of *Babesia caballi* in the culture medium [16]. Similarly, iron chelators, such as deferoxamine (DFO), mitochondrially targeted deferoxamine (mitoDFO) and 1-(N-acetyl-6-aminohexyl)− 3-hydroxy-2 methylpyridin-4-one (CM1), limit the availability of iron to the parasite and have been shown to inhibit *Babesia* growth in vitro [13,17].

However, iron is generally present in forms that are not readily bioavailable. Besides this, acquisition of iron by parasites is challenging due to host defense mechanisms that sequester iron to restrict pathogen growth, which is known as “nutritional immunity” [6,18]. To counteract this, *Babesia* like other protozoan parasites (*Plasmodium* and *Toxoplasma*), must actively acquire iron from its host. In Apicomplexa, iron uptake generally occurs via two major mechanisms: (1) direct acquisition of host-derived free iron through transporter-mediated uptake, and (2) uptake of iron bound to host proteins, such as transferrin, through receptor-mediated internalization followed by protein degradation and iron release [3,6]. A third possible mechanism involves the import of iron complexed with high-affinity ligands, such as heme, via dedicated transporters. This pathway requires the intracellular release of iron, which is often achieved by heme oxygenase mediated cleavage of the tetrapyrrole ring, an enzyme that is likely not functionally active in *Plasmodium* and *Babesia* species [19]. *Babesia* parasites replicate within red blood cells, a hemoglobin-rich environment and potential reservoir of heme-derived iron, and are presumed to encounter multiple host iron sources. Unlike other intraerythrocytic parasites such as *Plasmodium falciparum*, *Babesia* spp. form only a transient parasitophorous vacuole during erythrocyte invasion and do not retain a permanent vacuolar compartment during asexual intraerythrocytic development [20]. In addition, they do not form hemozoin, a crystalline product resulting from the condensation of free heme released during hemoglobin digestion. These distinctions imply a fundamentally different iron metabolism strategy, possibly involving direct uptake of host-derived iron or heme across the parasite membrane. Yet, the precise nature of the iron sources accessible to *Babesia*, as well as the molecular mechanisms underlying their acquisition and utilization, remain largely unresolved. Collectively, these suggest that *Babesia* has evolved specialized iron acquisition and regulatory pathways. A deeper understanding of these mechanisms may not only elucidate key aspects of *Babesia* biology but also identify novel molecular targets for therapeutic intervention aimed at disrupting iron homeostasis in the parasite [16,17].

## Methods

### Parasite culture

*B. divergens* 2210A G2 was maintained in a suspension of bovine erythrocytes sourced from a parasite-free cow, confirmed through culture testing (AnalytiChem Netherlands B.V.), following the methodology outlined by Jalovecka et al. (2016). Cultures were grown in RPMI 1640 medium (Lonza, Switzerland) enriched with 50 μg/ml gentamicin and 0.25 μg/ml amphotericin B (both from Merck), along with 20% heat-inactivated fetal calf serum (Capricorn), which was treated at 56 °C for 30 minutes prior to use. The incubation was conducted at 37 °C under a humidified atmosphere with 5% CO^2^ [21]. To visualize parasitemia, infected erythrocyte smears were fixed and stained using the Diff-Quik kit, adhering to the manufacturer's protocol.

### Merozoite purification

Infected red blood cells (iRBCs) from cultures exhibiting a parasitemia level of 20% or higher were collected by centrifugation at 600 × g for 5 minutes. The resulting pellet was resuspended in 1 × PBS to achieve a 10-fold cells concentration. Subsequently, the suspension was manually filtered twice at room temperature using Isopore filter units (1.2 μm pore size, 25 mm diameter). The filtered cells were centrifuged at 2000 × g for 5 minutes and then washed once with an isolation buffer containing 20 mM HEPES, 1.8 mM CaCl_2_, 5.3 mM KCl, 44 mM NaHCO_3_, 110.3 mM NaCl, and 11 mM D-glucose. Filtration was performed gently using a 20 mL syringe, maintaining a flow rate of approximately 5 mL/min. Following filtration, the viability of free merozoites used in the uptake assays was evaluated after 1 h of incubation at 37°C. Fluorescence microscopy was performed using MitoTracker Red to visualize parasites with active mitochondria and SYTOX Green to identify non-viable, membrane-compromised parasites. Viability was additionally quantified by flow cytometry using SYTOX Green staining, with both independent preparations showing >92% viable merozoites (S1 Fig).

### Iron uptake

Pelleted *B. divergens* merozoites were incubated at 37 °C for 1 hour in isolation buffer supplemented with 2 μM ^55^Fe(II)-ascorbate, 2 μM ^55^Fe(III)-citrate or 2 μM ^55^Fe-transferrin. To terminate iron uptake and remove extracellular nonspecifically associated iron, ethylenediaminetetraacetic acid (EDTA), a strong metal chelator commonly used to sequester free iron ions and prevent further uptake [22], was added post-incubation to a final concentration of 1 mM. Following treatment, cells were washed three times with isolation buffer to remove residual extracellular and surface-bound radioisotope. Protein concentration was determined using the BCA protein assay kit (Sigma-Aldrich, USA). For normalization, protein concentrations were adjusted to equivalent levels across all samples prior to subsequent analyses.

The radioactivity associated with the cells was quantified via liquid scintillation counting (Hidex Triathler, USA) after addition of liquid scintillation cocktail Ultima Gold LLT (PerkinElmer, USA).

Proteins containing radioactive iron were resolved using the Novex Native PAGE Bis–Tris Gel system (4–16%; Invitrogen, USA) as described by Schamel (2008). After electrophoresis, gels were vacuum-dried for 2 hours at 80°C and subjected to autoradiography using a tritium-sensitive phosphor screen (BAS-IP TR 2025 E) and Typhoon FLA (GE Healthcare). All experiments were independently replicated three times to ensure reproducibility.

### Ferric reductase activity

To assess the presence of a reductive iron uptake pathway in *B. divergens*, isolated parasites were incubated with ^55^Fe(III)-citrate in both the presence and absence of BPS, a selective chelator of ferrous iron. Incubations were performed for 1 hour either at 37 °C or on ice in an isolation buffer. The medium was supplemented with 1 μM ^55^Fe(III)-citrate, with or without 0.2 mM BPS. Post-incubation, cells were subjected to three washes via centrifugation using the same buffer to eliminate unbound extracellular iron. The level of iron internalized or associated with the cells was quantified through liquid scintillation counting.

Ferric reductase activity was measured using a ferrozine-based colorimetric assay. Samples were incubated in isolation buffer containing 1.3 mM ferrozine and 0.5 mM Fe(III)-citrate at 37°C in the dark. Two types of controls were included: (1) samples without Fe-citrate, and (2) samples without cells. After 3 h of incubation, cells were centrifuged, and the change in absorbance at 535 nm in the supernatant, corresponding to formation of the ferrozine–Fe(II) complex, was measured using a UV-2600 UV–Vis spectrophotometer.

### Apo-transferrin uptake

To investigate the effect of transferrin on *B. divergens* proliferation, parasites were cultured in vitro under four conditions: iRBCs alone (control), iRBCs supplemented with 6 mg/mL apo-transferrin, iRBCs supplemented with 15 mg/mL apo-transferrin and 15TF + 2 µM Fe(III)-citrate. Cultures were initiated at 2% parasitemia and maintained in RPMI 1640 medium supplemented with 25 mM HEPES, 11.5 mg/l hypoxanthine, 2.42 mM sodium bicarbonate and 4.31 mg/ml AlbuMAX II, pH 6.75, supplemented with 50 μg/ml gentamicin, and 0.25 μg/ml amphotericin B. All cultures were incubated at 37 °C in a humidified 5% CO_2_ atmosphere. Parasitemia levels were monitored every 48 hours over a 192-hour period by preparing Giemsa-stained thin blood smears. The percentage of iRBCs was determined by microscopic examination of at least 1,000 erythrocytes per slide. All experiments were conducted in triplicate, and values were plotted as mean ± standard deviation.

### Statistical analysis

All experiments were performed independently at least three times. Data are presented as mean ± standard deviation (SD). Prior to statistical comparisons, data were evaluated for normal distribution and equality of variances to ensure the appropriateness of parametric analyses. Statistical analyses were performed using GraphPad Prism 10 software. Comparisons between two groups were conducted using an unpaired two-tailed Student’s *t*-test, while comparisons among multiple groups were analyzed by one-way analysis of variance (ANOVA). Differences were considered statistically significant at *p* < 0.05.

### Immunogold transmission electron microscopy

Cell pellets of *Babesia*-infected red blood cells were fixed in a solution containing 4% formaldehyde and 0.1% glutaraldehyde in 0.1 M HEPES (Sigma-Aldrich) for 1 h at room temperature. After fixation, the cells were washed in 0.1 M HEPES, embedded in 10% gelatin (Serva Electrophoresis), and transferred into 2.3 M sucrose for cryoprotection for 72 h at 4 °C. The gelatin-embedded pellets were cut into small blocks, mounted on flat copper stubs, and plunge-frozen in liquid nitrogen. Ultrathin cryosections (70 nm) were prepared using a UC6/FC6 cryo-ultramicrotome (Leica Microsystems) at −100 °C. Sections were collected with a drop of 1% methyl cellulose / 1.05 M sucrose and transferred onto formvar-coated nickel grids.

For immunolabeling, sections were incubated for 1 h in blocking buffer (BB) consisting of 1% fish skin gelatin (Sigma-Aldrich) in 0.1 M HEPES, followed by incubation with the primary antibody (goat anti-transferrin) diluted 1:30 for 30 min at room temperature. After washing with BB, sections were incubated with a secondary antibody conjugated to 5 nm gold nanoparticles (rabbit anti-goat, batch 7443; BBI) diluted 1:50 in BB for 30 min at room temperature. Negative-control samples were processed in parallel with all staining steps except incubation with the primary antibody, allowing assessment of nonspecific secondary antibody binding. Finally, sections were washed in HEPES and distilled water, then contrasted and embedded using a mixture of 2% methyl cellulose and 3% aqueous uranyl acetate (9:1). Cryosections were examined using a JEOL 1400-F transmission electron microscope equipped with an XAROSA 20 MP CMOS camera (EMSIS GmbH) operated at 120 kV.

### Comparative proteomics and structural predictions

#### Parasite cultivation and sample preparation.

*B. divergens* cultures (2% initial parasitemia) were maintained under normal conditions and iron-chelating conditions with final concentration 10 μM deferoxamine (DFO) in 75 cm^2^ aerobic flasks for 48 hours. Parasites were harvested by centrifugation (2000 × g, 5 min, 4 °C) and washed with 1 × PBS, pH 7.4. Experiments were performed using three biological replicates.

#### Protein extraction and digestion.

Parasite pellets were lysed in 2% SDC in 100 mM triethylammonium bicarbonate (TEAB) buffer, heated at 95 °C for 5 min, and sonicated. Protein concentrations were measured via BCA assay (Sigma-Aldrich, USA). For digestion, 30 µg protein was mixed with TEAB buffer containing 2% SDC, 50 mM chloroacetamide, and 10 mM tris(2-carboxyethyl)phosphine (TCEP). After boiling, samples were combined with SP3 beads and 100% ethanol, washed with 80% ethanol, and digested overnight at 37 °C using trypsin. Peptides were acidified with 1% trifluoroacetic acid and desalted using in-house C18 stage tips [23].

#### nLC-MS/MS analysis.

Peptides were analyzed using a Thermo Orbitrap Ascend system with a C18 UHPLC column (Ion Opticks). Mobile phases were water or acetonitrile with 0.1% formic acid. A linear gradient from 4–35% mobile phase B over 60 min was used. Data-dependent acquisition covered 350–1400 m/z at 120K resolution. MS/MS was performed with quadrupole isolation (1.6 Da), HCD fragmentation (NCE 30), and ion trap detection. Cycle time was 1.5 s with dynamic exclusion of 30 s.

#### Data processing.

Data were analyzed with MaxQuant v2.4.13.0, using Andromeda against *B. divergens* and *Bos* *taurus* databases (UniProt, June/May 2023). Enzyme specificity included Arg/Lys cleavages, permitting up to two missed cleavages. Carbamidomethylation (C) was fixed; N-terminal acetylation and methionine oxidation were variable. A 1% FDR was applied at peptide/protein levels. Quantitative analysis employed the “match between runs” option (0.7 min retention window), followed by downstream analysis in Perseus v1.6.15.0. The mass spectrometry proteomics data have been deposited to the ProteomeXchange Consortium via the PRIDE [24] partner repository with the dataset identifier PXD081904.

#### Homology and structural predictions.

Protein sequence homology was assessed using PSI-BLAST [25] against the NCBI non-redundant database, selecting hits with >80% identity and E-values < 1e^-24^. Protein-heme interaction models were generated via AlphaFold3 [26], using heme as the ligand. Visualization and analysis were conducted in PyMOL.

#### Statistical analysis.

Unless stated otherwise, two-tailed Student’s t-test with two-sample equal variance was used to determine the p-values. Unless stated otherwise, all experiments were performed in three or more independent replicates, meaning parallel experiments on independently cultivated cultures.

LFQ data from MaxQuant were used (version 2.4.13.0) [27]. Data were further analyzed using Perseus software (version 1.6.15.0, Computational Systems Biochemistry) [28]. Free merozoites and infected red blood cell datasets were analyzed separately. Filtering steps included the removal of hits to the reverse database and potential contaminants. A 1% FDR was applied at peptide/protein levels and proteins identified by a single peptide that has a score lower than 40 were excluded from further analysis. Subsequently, t-tests were calculated for treated and control group comparisons and the significance level was determined using permutation-based FDR.

Functional annotation of *B. divergens* proteins was carried out using PiroplasmaDB (Release 68), a component of the VEuPathDB Bioinformatics Resource Center [29].

## Results

### Reductive iron uptake enables integration of iron into protein complexes in *Babesia divergens*

To identify the mechanisms by which isolated intraerythrocytic *B. divergens* parasites utilize iron, we quantified the uptake kinetics of radioactive iron supplied in different forms. Among the tested substrates, transferrin exhibited the highest uptake rate, reaching 6.76 ± 0.30 nmol Fe·min^-1^·g^-1^ protein, suggesting it serves as a natural iron source for the parasite. The ^55^Fe(II)-ascorbate uptake rate was 3.73 ± 0.40 nmol Fe·min^-1^·g^-1^ protein, whereas the uptake from ^55^Fe(III)-citrate was significantly lower at 2.47 ± 0.20 nmol Fe·min^-1^·g^-1^ protein (Fig 1A). These results are indicative of a preferential ferrous iron assimilation pathway and suggest the involvement of a ferric reductase activity in the mobilization of iron from ferric iron complexes. Furthermore, incubation of the parasites at 4 °C led to a marked decline in iron uptake by 44.4% for transferrin and 48.7% for Fe(II), Fig 1A, corroborating the notion that iron acquisition in *B. divergens* is predominantly an energy-dependent, active transport process. This temperature sensitivity supports the involvement of membrane-associated transporters or enzymatic systems that require metabolic activity for efficient iron import, rather than passive diffusion.

**Fig 1:**
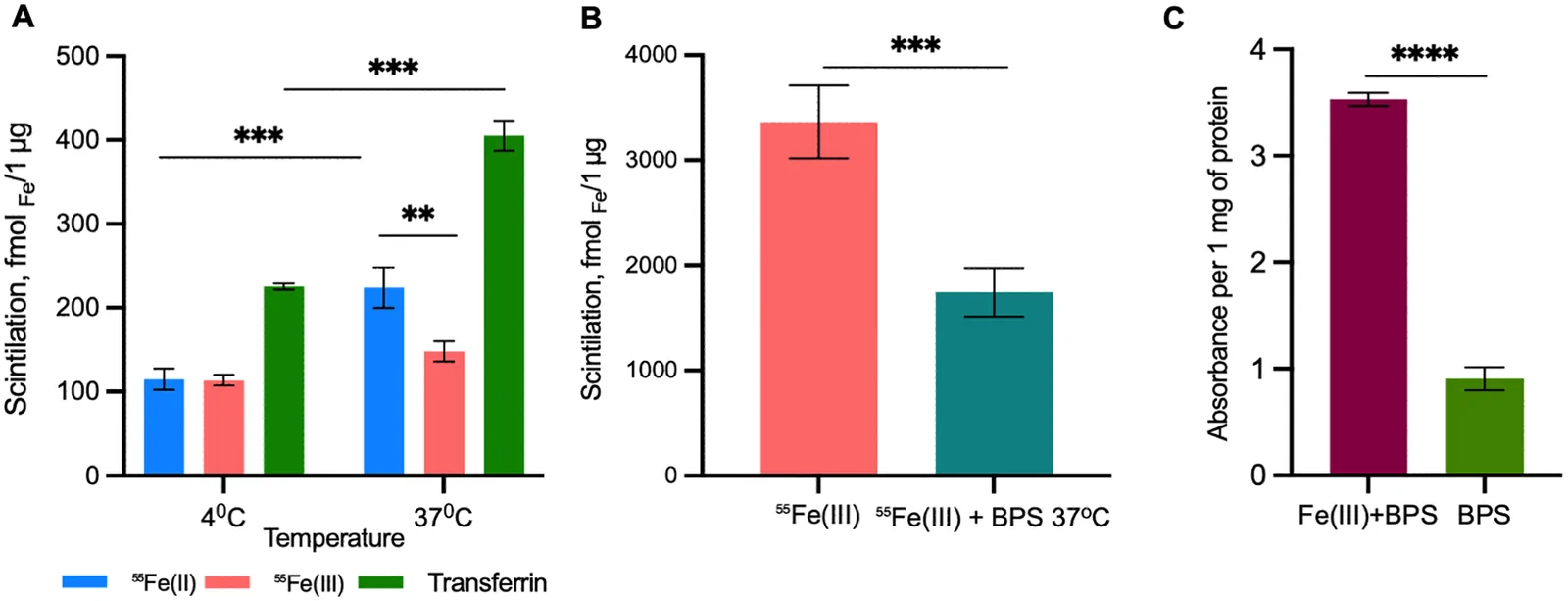
Iron radioisotope uptake from different sources and ferric reductase activity in *Babesia divergens* merozoites. **(A)** Uptake of radioactive iron is significantly increased at 37 °C compared to 4 °C for three tested substrates after 1 hour incubation. ^55^Fe-transferrin showed the highest uptake, followed by ^55^Fe(II)-ascorbate and ^55^Fe(III)-citrate, indicating a preference for ferrous iron and supporting a temperature-dependent, active transport mechanism. Data are presented as mean ± SD from three independent experiments (**p < 0.01, ***p < 0.001). **(B)** Uptake of ferric iron from ^55^Fe(III)-citrate was significantly inhibited by the ferrous iron chelator BPS, consistent with a requirement for Fe(III) reduction prior to transport. Data are shown as mean ± SD, representative of three independent experiments (***p < 0.001). **(C)** Extracellular ferric reductase activity of *Babesia divergens* merozoites demonstrated by a BPS-based colorimetric assay, showing significant reduction of ferric to ferrous iron in the presence of intact cells. Data are shown as mean ± SD, representative of three independent experiments (****p < 0.0001).

To further elucidate whether *B. divergens* utilizes a reductive pathway for iron acquisition, we conducted uptake assays using ^55^Fe(III)-citrate in the presence or absence of the ferrous iron-specific chelator bathophenanthroline disulfonate (BPS). Cultures incubated with 0.2 mM BPS at 37 °C exhibited a significant reduction (~49%) in cell-associated radioactivity relative to untreated controls (p < 0.001; Fig 1B). Given that BPS selectively binds Fe(II), thereby preventing its cellular uptake, these data strongly support the hypothesis that ferric iron must undergo reduction to its ferrous state prior to internalization.

To provide additional support for our findings, ferric reductase activity of *B. divergens* free merozoites was confirmed using a BPS-based colorimetric assay. The cells exhibited a ferric reductase activity of 2.14 ± 0.23 pmol/min per 10^6^ cells when Fe(III)-citrate was used as the iron source (Fig 1C). This activity reflects the formation of the BPS–Fe(II) complex resulting from extracellular reduction of ferric iron. These results confirm that *B. divergens* free merozoites possess measurable extracellular ferric reductase activity under the tested conditions.

To assess whether internalized iron is functionally incorporated into parasite macromolecular structures, we performed blue native PAGE (BN-PAGE) analysis on lysates from merozoites incubated with either ^55^Fe(III)-citrate or ^55^Fe(II)-ascorbate. Autoradiography of the native gels (S2 Fig) revealed that ^55^Fe-associated radioactivity localized to discrete high-molecular-weight protein complexes, predominantly in the ^55^Fe-treated samples incubated at physiological temperature. These findings indicate that iron is internalized and even more actively integrated into native multiprotein assemblies, supporting its incorporation into metabolically relevant iron-binding complexes.

These findings collectively demonstrate that *B. divergens* employs a sequential mechanism of iron acquisition, wherein ferric iron is initially reduced to the ferrous state prior to cellular uptake. This stepwise process facilitates the effective utilization of diverse iron substrates, reflecting the parasite’s metabolic adaptability to varying iron availability.

### Utilization of transferrin-bound iron supports *Babesia divergens* propagation within host erythrocytes

Following uptake experiments indicating transferrin as a potential physiological iron source for intraerythrocytic *B. divergens*, we examined whether apo-transferrin influences parasite proliferation through competitive sequestration of bioavailable iron. Cultures exposed to different concentrations of apo-transferrin demonstrated dose-dependent growth inhibition. While 6 mg/mL apo-transferrin had negligible impact on parasite proliferation, a concentration of 15 mg/mL apo-transferrin significantly (p < 0.0001) suppressed parasitemia at 48 hours, reducing parasite burden by approximately one half (~49%) (Fig 2A and 2B). Supplementation of the culture with Fe(III)-citrate rescued parasite growth inhibited by apo-transferrin, restoring parasitemia to levels comparable to the untreated control. This inhibitory effect likely reflects apo-transferrin’s capacity to interfere with iron acquisition dynamics by impeding transferrin-dependent iron delivery pathways. These observations underscore the functional relevance of transferrin in *B. divergens* iron metabolism and suggest that iron sequestration strategies may effectively compromise parasite viability.

**Fig 2:**
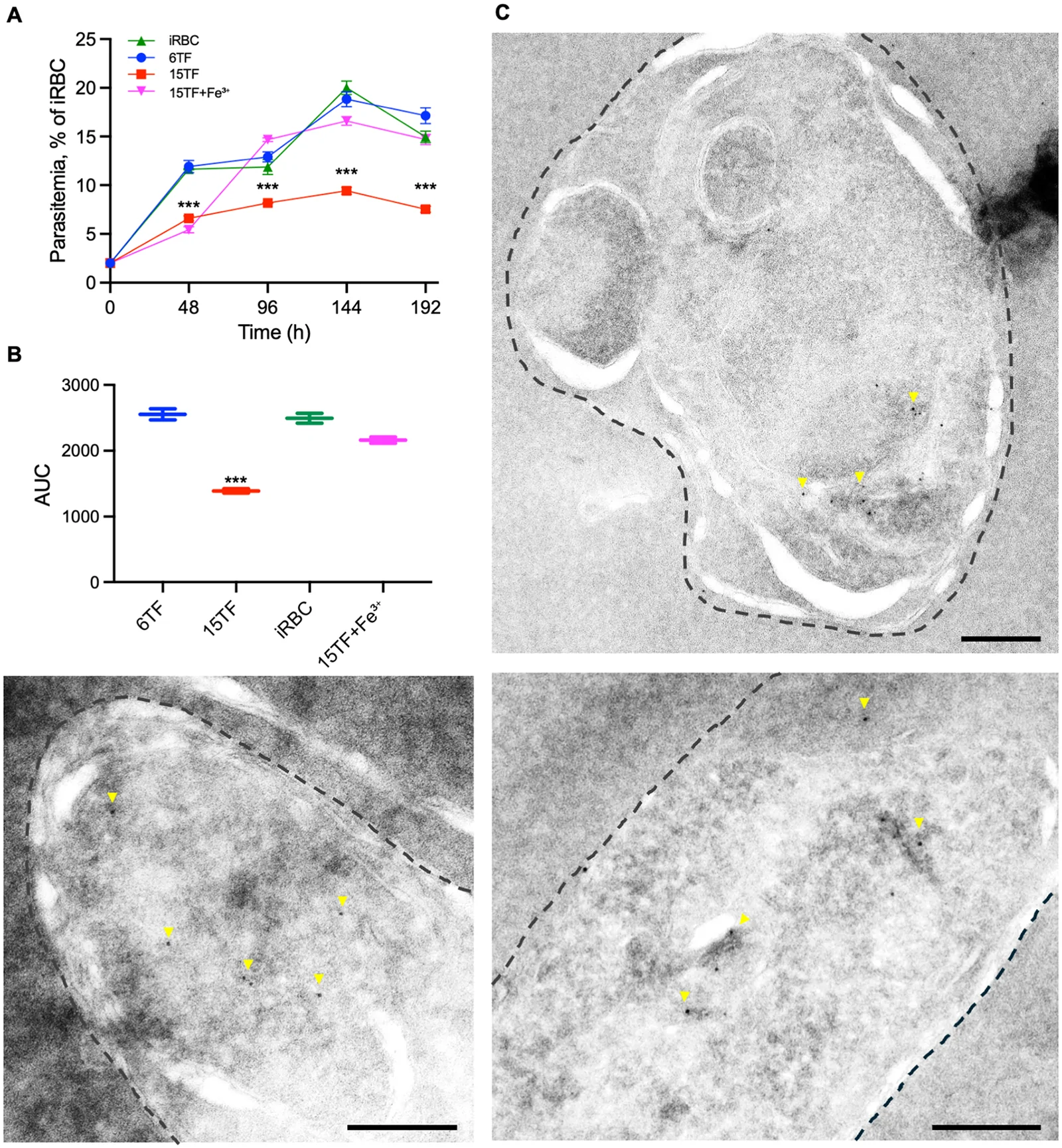
Uptake and utilization of apo-transferrin by *Babesia divergens.* **(A)** Growth of *B. divergens* in *in vitro* cultures supplemented with different concentrations of apo-transferrin. Parasitemia was monitored over time in cultures containing 6 mg/mL apo-transferrin (6TF, blue), 15 mg/mL apo-transferrin (15TF, red), 15 mg/mL apo-transferrin and 2 µM Fe(III)-citrate (15TF + Fe^3+^, magenta) and control infected red blood cells without added apo-transferrin (iRBC, green). The 15TF displayed decreased parasitemia levels in comparison to 6TF and iRBC control conditions, indicating a dose-dependent effect of apo-transferrin on parasite proliferation. **(B)** AUC analysis of parasite growth over the 192-hour growth period. Boxplots represent mean AUC values ± SD. Asterisks indicate significantly lower mean AUC values of 15TF compared to the iRBC control (***p < 0.001). AUC = area under curve. **(C)** Transmission electron microscopy (TEM) images of *B. divergens*-infected red blood cells incubated with immunolabeled transferrin show parasite internal compartments containing transferrin (yellow arrowheads), confirming internalization of the glycoprotein, scale bar 200 nm.

The inhibitory effect of apo-transferrin on parasite proliferation likely reflects its internalization by *B. divergens* through a yet-uncharacterized uptake pathway adapted for acquisition of iron-loaded transferrin. However, as apo-transferrin lacks ferric iron content, its internalization fails to deliver bioavailable iron to the parasite. Thus, this form of iron depletion compromises essential iron-dependent cellular processes, including mitochondrial respiration, nucleic acid synthesis, and cell cycle progression. The resultant intracellular iron insufficiency impairs metabolic function and halts proliferation.

To corroborate the internalization of transferrin by *B. divergens*, immunogold transmission electron microscopy (immuno-TEM) was employed. Ultrastructural analyses revealed definitive localization of transferrin within the cytoplasm and organellar compartments of intraerythrocytic stages of *B. divergens* (Figs 2C and S3). These findings provide direct evidence supporting transferrin uptake and complement the biochemical data, collectively substantiating the parasite’s capacity for internalizing and utilizing transferrin-bound iron through an active acquisition pathway.

### Proteomic analysis reveals stage-specific responses to iron chelation in *Babesia divergens* blood stages

To investigate the effect of iron chelation on *B. divergens* blood-stage parasites, we performed label-free quantitative proteomic analysis of free merozoites and infected red blood cells (iRBCs) from cultures maintained in the presence or absence of the iron chelator deferoxamine (DFO) (Fig 3A).

**Fig 3:**
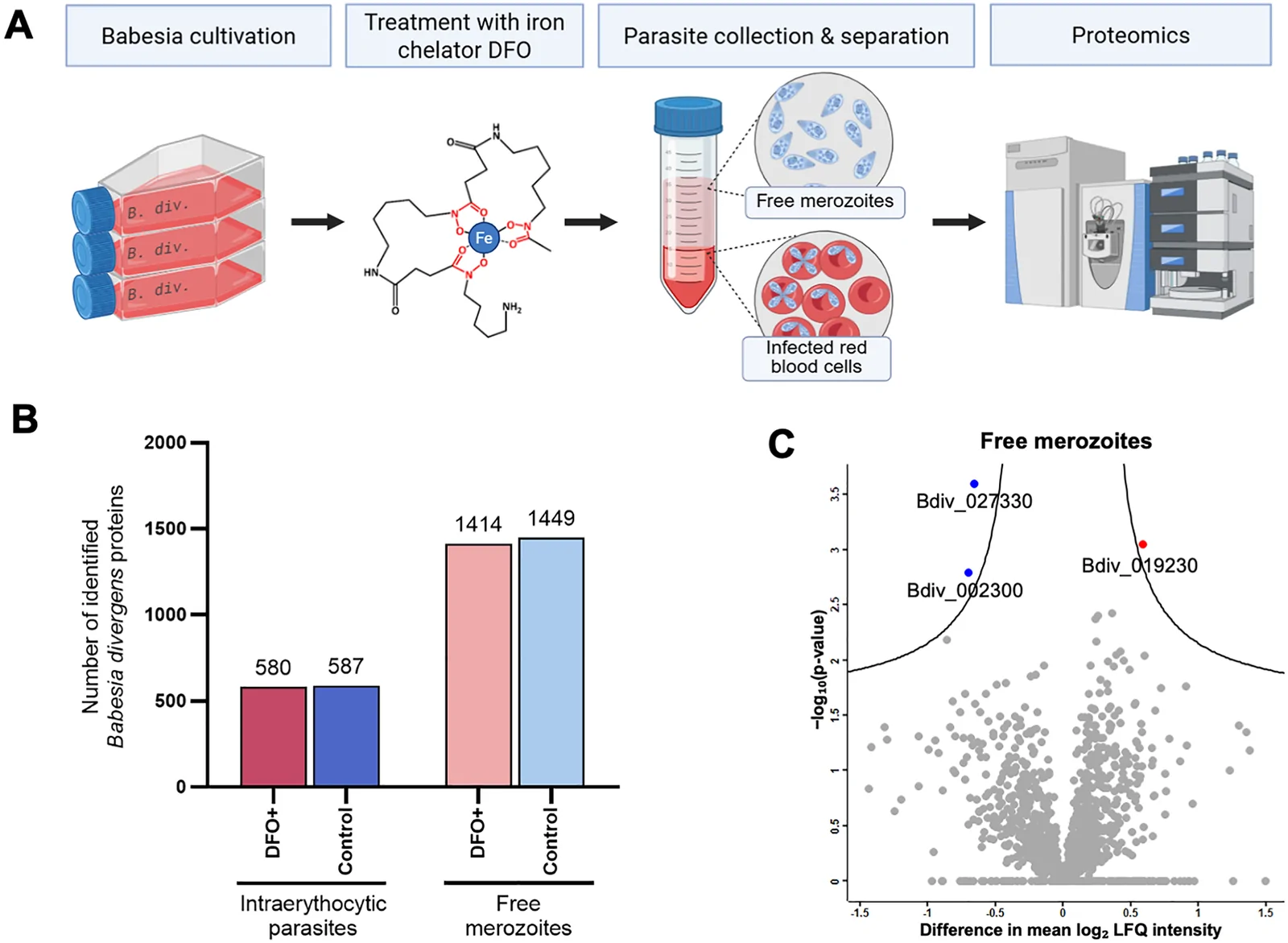
Proteomic response of *Babesia divergens* to iron depletion induced by deferoxamine treatment. **(A)** Schematic overview of the experimental workflow. Cultured *B. divergens* parasites were maintained under control conditions or treated with the iron chelator deferoxamine (DFO), collected and separated into free-merozoite and intraerythrocytic-parasite fractions for proteomic analysis. Created with BioRender.com. **(B)** Number of *B. divergens* proteins identified in control and DFO-treated intraerythrocytic-parasite and free-merozoite fractions. A protein was considered identified in a fraction when it was detected in at least one of the three biological replicates. **(C)** Volcano plots comparing protein abundance between DFO-treated and control samples in free merozoites. The analysis was performed using normalized log_2_-transformed LFQ intensities without missing-value imputation. Curved significance boundaries were determined using a permutation-based false discovery rate (FDR) of 0.05, S_0_ = 0.1, and 250 randomizations. Proteins significantly increased or decreased in abundance following DFO treatment are shown in red and blue, respectively. Selected significant proteins are labelled with their PiroplasmaDB identifiers.

Overall, 1,455 *B. divergens* proteins were identified in the free-merozoite samples, whereas 590 parasite proteins were identified in the intraerythrocytic-parasite samples (Fig 3B; S1 and S2 Tables). The lower number of parasite proteins detected in the intraerythrocytic fraction was likely influenced by the high abundance of host haemoglobin and other erythrocyte proteins, which can limit the detection of low-abundance parasite proteins [30].

Differences in protein abundance between DFO-treated and control samples were evaluated using two-sample Student’s t-tests with a permutation-based false discovery rate (FDR). The resulting volcano plots revealed statistically significant changes in protein abundance in free merozoites, whereas no proteins reached the significance threshold in intraerythrocytic parasites (Fig 3C; S1 and S2 Tables).

In free merozoites, three proteins showed statistically significant changes in abundance following DFO treatment (Fig 3C). Bdiv_019230 increased in abundance, whereas the hypothetical protein Bdiv_002300 and the putative serine hydroxymethyltransferase Bdiv_027330 decreased in abundance. Bdiv_019230 was predicted to encode a multipass membrane protein. PSI-BLAST identified high-confidence homologues across several *Babesia* species, including proteins annotated as putative integral membrane proteins, nucleoside transporters, and a CLN3-domain-containing protein. These results support a possible transporter-like function but do not establish either its substrate specificity or its direct involvement in iron transport.

Bdiv_027330 is annotated as a putative serine hydroxymethyltransferase, an enzyme linking serine-glycine metabolism with one-carbon-unit production and nucleotide biosynthesis. Its decreased abundance may reflect reduced biosynthetic activity in DFO-treated merozoites, although the present results do not establish whether this effect is directly regulated by iron availability or represents a broader response to DFO-induced metabolic stress. Bdiv_002300 remains functionally uncharacterized and its relationship to iron homeostasis cannot currently be determined.

## Discussion

*Babesia* spp*.* are obligate intracellular protozoan parasites that invade and replicate within host erythrocytes. While hemoglobin constitutes a major and readily accessible source of iron within host erythrocytes, the potential for *Babesia* spp. to acquire iron from alternative extraerythrocytic sources cannot be excluded. Notably, the absence of a parasitophorous vacuole, a key feature distinguishing *Babesia* from other apicomplexan parasites, combined with the toxicity of heme and the absence of hemozoin formation, suggests that these parasites may have evolved specialized mechanisms to access and exploit extracellular iron sources. Interestingly, Apicomplexan parasites, including *Babesia*, often lack canonical mammalian iron-handling proteins, suggesting that they have evolved unique mechanisms to regulate iron acquisition, storage, export and detoxification [3].

Extracellular iron in mammalian hosts is primarily sequestered by high-affinity binding proteins such as transferrin and lactoferrin, which maintain extremely low concentrations of free iron in plasma and thereby create a restrictive environment for pathogen proliferation. Similar to other apicomplexan parasites that have evolved receptors or scavenging mechanisms to access host-bound iron, *Babesia* spp. likely exploit extracellular iron sources [3,30]. In this study, we demonstrate that *B. divergens* is capable of utilizing transferrin-bound iron, a major physiological iron source in the host. This was visually confirmed by immunogold TEM, which revealed the presence of transferrin within the cytoplasm and organellar compartments of intraerythrocytic parasites. The marked reduction in transferrin uptake at low temperatures suggests that *B. divergens* employs an active, energy-dependent process for host-derived iron acquisition. Importantly, the observed dose-dependent suppression of *B. divergens* growth by apo-transferrin suggests that iron availability is a limiting factor for parasite proliferation. At elevated concentrations, apo-transferrin likely disrupts iron homeostasis by restricting access to functional iron sources. Apicomplexan parasites do not possess canonical transferrin receptors like those found in mammals [31]. However, *T.* *gondii* also employs host protein scavenging as an iron acquisition strategy. Tachyzoites have been shown to endocytose host proteins including transferrin receptor 1, transferrin, ferritin heavy and light chains, and clathrin light chain [32]. Additionally, *T.* *gondii* and other Apicomplexans express lactoferrin-binding proteins that enable utilization of holo-lactoferrin as an iron source, a crucial adaptation in iron-restricted environments like mucosal tissues [33]. These insights raise the possibility that *Babesia* may encode yet-undescribed transporters or receptor-like proteins to mediate transferrin and/or lactoferrin uptake. In *P.* *falciparum*, it has been functionally proved that the plasma membrane transporter PfE140 mediates iron uptake and may represent a functional analog to yet-unidentified surface transporters in *Babesia* that facilitate iron import [34].

In *P*. *falciparum*, mitochondrial import is facilitated by the mitoferrin homolog PfMRS3, essential for ISC and heme biosynthesis [34]. Additionally, mitochondrial ABC transporters such as ATM1 and ABCB7, which mediate the export of [ISC] intermediates in *T*. *gondii* and *P*. *falciparum* [35,36], may also be functionally conserved in *Babesia*, although this remains to be experimentally validated.

Iron chelation induced a coordinated remodeling of metabolic and replication-associated pathways in free merozoites, consistent with a transition from a proliferative to a stress-adapted physiological state.

Three proteins were significantly altered in free merozoites following DFO treatment. Bdiv_019230 is predicted to be a multipass membrane protein and our comparative sequence analysis identified homologues in other *Babesia* species; however, neither its substrate specificity nor a direct role in iron transport has been established. Bdiv_027330 is associated with serine-glycine and folate-dependent one-carbon metabolism, which supplies one-carbon units required for nucleotide biosynthesis and cellular proliferation. In *Plasmodium*, serine hydroxymethyltransferase has been biochemically characterized and shown to be indispensable for parasite development [37,38]. Its altered abundance may therefore reflect metabolic adaptation to iron deprivation rather than direct participation in iron uptake. Bdiv_002300 remains functionally uncharacterized. Collectively, these proteins represent statistically supported components of the DFO response, but their mechanistic links to iron homeostasis require experimental validation.

Although Bdiv_001180 showed a consistent trend towards increased abundance following DFO treatment in both free merozoites and intraerythrocytic parasites (2.6-fold and 4.65-fold, respectively), neither change reached the permutation-based FDR significance threshold. Therefore, Bdiv_001180 cannot be classified as differentially abundant or iron-responsive. Nevertheless, its predicted surface localization and the reported function of a distant fungal homologue as a haem/haemoglobin receptor [39,40] make it a hypothesis-generating candidate for future investigation. Exploratory structural modelling is presented in S4 Fig; however, targeted biochemical and genetic studies will be required to establish whether Bdiv_001180 has any role in haem acquisition or the response to iron limitation.

Among the proteins absent after DFO treatment in free merozoites was a putative thioredoxin (Bdiv_001970c), a core component of the parasite's antioxidant defense system. Because *Babesia*, like other apicomplexans, relies heavily on the Trx/TrxR/NADPH system to counter host-derived oxidative stress in the relative absence of catalase and glutathione peroxidase [41], loss of thioredoxin expression under iron restriction likely reflects a downscaling of redox defense capacity rather than a compensatory response. This may be functionally consequential: *B. microti* thioredoxin reductase (BmiTrxR) possesses a C-terminal redox center built from two cysteines rather than the cysteine-selenocysteine motif found in mammalian TrxR [42], a structural divergence already being exploited for the design of *Babesia*-selective TrxR inhibitors. Given that TrxR inhibition in *B. microti* has been shown to elevate ROS, lipid peroxidation and protein carbonylation [43], the DFO-induced suppression of thioredoxin we observed may similarly compromise redox defense in free merozoites, and, together with the parasite-specific architecture of the Trx system, underscores this pathway's relevance both to iron-restriction biology and as a potential therapeutic vulnerability.

It is plausible that *Babesia* may access other intracellular iron sources such as cytosolic iron pools through parasite-derived enzymes or membrane-bound transporters, as observed in other protozoan parasites. For instance, *P*. *falciparum* utilizes the DMT1 homolog PfDMT1 (also known as PfNRAMP) localized to the digestive vacuole membrane to export labile Fe^2+^ released from hemoglobin degradation [44]. Although the presence of a food vacuole in *Babesia* has not yet been clearly demonstrated, it encodes homologs of DMT1 [3], suggesting that similar transporters may facilitate direct ferrous iron import across the parasite plasma membrane from the host cytosol. In *P*. *falciparum*, vesicular iron sequestration is mediated by PfVIT, with PfZIPCO mobilizing stored iron under scarcity [45].

Collectively, these findings support a model in *B*. *divergens* where iron acquisition proceeds through a sequential, multi-route system: initial ferric reduction (possibly via parasite-encoded reductases), active transport of ferrous iron and the utilization of host-derived transferrin through an uncharacterized yet functional internalization pathway that may involve surface or endocytic transporters. The parasite's ability to exploit both inorganic and transferrin-bound iron sources underlines its adaptive capacity to host environments, offering potential targets for therapeutic intervention by disrupting iron homeostasis [46]. The statistically significant increase in Bdiv_019230 identifies this conserved multipass membrane protein as a candidate for functional investigation of parasite responses to iron restriction.

## Supporting information

S1 Fig Validation of the isolation procedure and viability of free merozoites of *Babesia divergens* used for iron uptake assays. **(A)** Representative Giemsa-stained smear of the isolated *B. divergens* free merozoites preparation following the optimized filtration and sedimentation protocol, demonstrating predominantly free parasites with minimal erythrocyte ghost contamination. **(B)** Assessment of parasite viability after 1 h incubation at 37°C using MitoTracker Red (viable parasites) and SYTOX Green (non-viable parasites). The vast majority of isolated parasites remained viable under the conditions used for the iron uptake assay. **(C)** Flow cytometric quantification of SYTOX Green-positive parasites from two independent isolations after 1 h incubation at 37°C, showing parasite viabilities of 92.6% and 93.4%. **(D)** Representative Giemsa-stained smear following the invasion assay demonstrating successful reinvasion of freshly isolated merozoites into erythrocytes after incubation.
(TIFF)

S2 Fig Association of iron radioisotope with protein complexes in *B. divergens* merozoites. **Free merozoites were incubated with ^55^Fe(III)-citrate at 37 °C and 4 °C.** Uninfected red blood cells processed identically to the parasite samples were included as a negative control. Native protein complexes were extracted using digitonin-based fractionation, followed by BN-PAGE (Blue Native PAGE, 4–16%). Protein bands containing radioactive iron were visualized by autoradiography. Distinct bands were detected primarily at 37 °C in the ^55^Fe(III) condition, suggesting specific incorporation of iron into native protein complexes, whereas no radioactive bands were detected in identically processed uninfected red blood cells, indicating that the detected signal is not associated with host erythrocyte proteins.
(TIFF)

S3 Fig Supporting data for Fig 2 (Uptake and utilization of apo-transferrin by *Babesia divergens*). Immunogold TEM controls performed without the primary antibody showed no gold labeling, confirming the absence of nonspecific binding by the secondary antibody.
(TIFF)

S1 Table Comparison of whole-cell proteomes of *Babesia divergens* merozoites under iron chelation with deferoxamine. The table presents proteomic profiling results of *B. divergens* merozoites cultured under deferoxamine (DFO) treatment. Proteins showing a fold change > 1.5 (upregulated under DFO treatment) or <–1.5 (downregulated under DFO treatment) were considered significantly regulated. Protein annotation was performed using PiroplasmaDB, with additional manual annotation of selected proteins as described in the Methods section. The experiment was conducted using three independent biological replicates.
(XLSX)

S2 Table Comparison of whole-cell proteomes of *Babesia divergens* infected red blood cells under iron chelation with deferoxamine. The table presents proteomic profiling results of *B. divergens* infected red blood cells cultured under deferoxamine (DFO) treatment. Proteins showing a fold change > 1.5 (upregulated under DFO treatment) or <–1.5 (downregulated under DFO treatment) were considered significantly regulated. Protein annotation was performed using PiroplasmaDB, with additional manual annotation of selected proteins as described in the Methods section. The experiment was conducted using three independent biological replicates.
(XLSX)

S4 Fig Exploratory AlphaFold3 prediction of Bdiv_001180 in complex with heme. **(A)** Structural model of Bdiv_001180 generated using AlphaFold3 [26] with heme included as a ligand (pink sticks). The predicted protein–heme complex achieved ipTM/pTM scores of 0.59/0.49, indicating low confidence in the predicted interaction. **(B)** Enlarged view of the region predicted by AlphaFold3 to interact with heme. In the model, the heme iron (orange sphere) is positioned near His126 within a five-helical bundle, while the heme propionate groups remain solvent exposed. Owing to the low confidence of the predicted complex, these features should be considered hypothesis-generating and require experimental validation.
(TIFF)

S1 Rawgel Original BN-PAGE autoradiographs corresponding to S2 Fig. Free *B. divergens* merozoites (FM) were incubated with ^55^Fe(III)-citrate at 37 °C and 4 °C. Uninfected red blood cells (RBC) processed identically were included as a negative control. Native protein complexes were extracted using digitonin-based fractionation and separated by Blue Native PAGE (BN-PAGE, 4–16%). Radioactive iron associated with native protein complexes was visualized by autoradiography.
(PDF)
