# Supplementary material for: Iron sourcing strategies and adaptive protein responses to iron deprivation in the intraerythrocytic parasite Babesia divergens
Source: PLoS Negl Trop Dis. 2026 Nov 4;20(11):e0014861. doi: 10.1371/journal.pntd.0014861 (PMC13759094; doi:10.1371/journal.pntd.0014861)
Supplement: Supplementary file 1 [file pntd.0014861.s007.pdf]

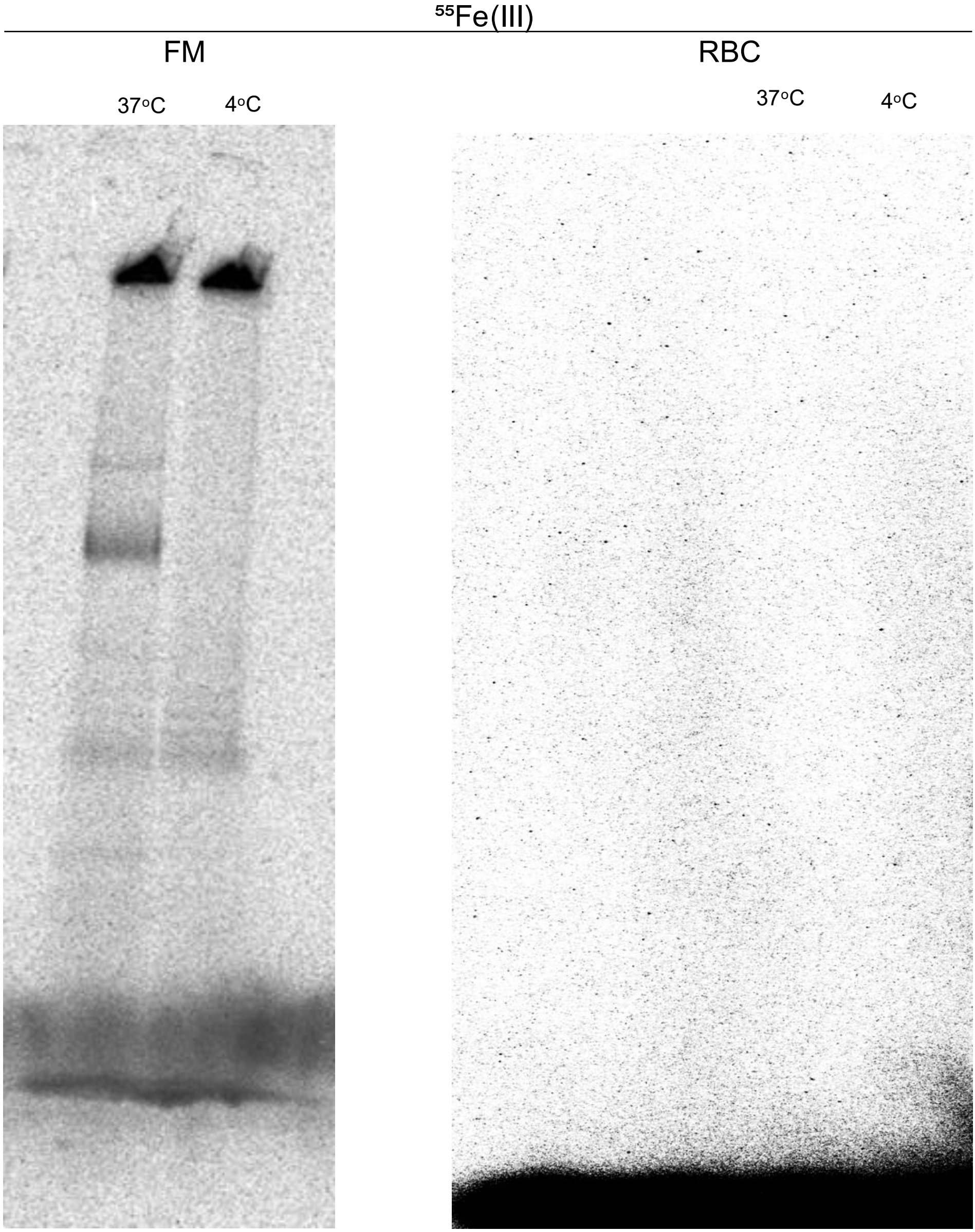
$^{55}Fe(III)$
FM
RBC
37°C
4°C
37°C
4°C
